# Genome Sequence of *Galleria mellonella* (Greater Wax Moth)

**DOI:** 10.1128/genomeA.01220-17

**Published:** 2018-01-11

**Authors:** Anna Lange, Sina Beier, Daniel H. Huson, Raphael Parusel, Franz Iglauer, Julia-Stefanie Frick

**Affiliations:** a Department for Medical Microbiology and Hygiene, Interfaculty Institute for Microbiology and Infection Medicine, University of Tübingen, Tübingen, Germany; b Algorithms in Bioinformatics, ZBIT Center for Bioinformatics, University of Tübingen, Tübingen, Germany; c Einrichtung für Tierschutz, Tierärztlichen Dienst und Labortierkunde, Eberhard Karls Universität Tübingen, Tübingen, Germany

## Abstract

The larvae of the greater wax moth, *Galleria mellonella*, are pests of active beehives. In infection biology, these larvae are playing a more and more attractive role as an invertebrate host model. Here, we report on the first genome sequence of *Galleria mellonella*.

## GENOME ANNOUNCEMENT

A ubiquitous pest of beehives, the greater wax moth causes severe damage due to its destructive way of feeding (1). As an invertebrate host model, it is used to study the virulence of different pathogens, such as methicillin-resistant *Staphylococcus aureus* (MRSA) (2), *Listeria monocytogenes* (3), and *Candida* spp. (4). So far, molecular biological studies of gene expression in *Galleria mellonella* have been based on a published transcriptome data set (5), and there was no genome sequence available. However, a genome sequence is crucial to enable homology studies between *Galleria mellonella* and human, mouse, and other model hosts. Here, we describe the first draft genome sequences available for *Galleria mellonella* (isolate FT-Tue), based on PacBio technology. Genomic DNA was extracted using the Qiagen Genomic-tip 100/G kit. Sequencing was performed at GATC Biotech AG (Constance, Germany) using PacBio long-read technology. A PacBio standard genomic library was sequenced on 22 single-molecule real-time (SMRT) cells. After subread filtering, this resulted in a total of 20,638,932,410 bases, 2,141,900 reads, an *N*_50_ read length of 13,454 bp, and a mean length of 9,635 bp. Our assembly of the genome produced 1,937 contigs comprising 578 Mbp, with an *N*_50_ of 952 kbp. The largest contig was 8.98 Mbp.

This first draft genome sequence will help to promote the use of *G. mellonella* as a replacement organism for vertebrates in biomedical research.

### Accession number(s).

This whole-genome shotgun project has been deposited at DDBJ/ENA/GenBank under the accession number NTHM00000000. The version described in this paper is version NTHM01000000.
